# Supplementary material for: Optimisation of Physical and Chemical Treatments to Control Browning Development and Enzymatic Activity on Fresh-cut Apple Slices
Source: Foods. 2020 Jan 9;9(1):76. doi: 10.3390/foods9010076 (PMC7022590; doi:10.3390/foods9010076)
Supplement: Supplementary file 1 [file foods-09-00076-s001.pdf]

Table S1. ANOVA table showing Total model ($p < 0.05$), main effects and interaction effect among the factors used in the experimentation

Factors		Enzymatic activity						Colour parameters at different air exposure time kept in the room temperature								
		PPO 0	PPO 30	PPO 60	POD 0	POD 30	POD 60	L* 0	a* 0	b* 0	L* 30	a* 30	b* 30	L* 60	a* 60	b* 60
Variety (D)		**< 0.001**	**< 0.001**	**< 0.001**	**< 0.001**	**< 0.001**	**< 0.001**	**0.001**	NS	**< 0.001**	**< 0.001**	NS	**0.003**	**0.005**	NS	**0.040**
Water source (a)		NS	**0.003**	**< 0.001**	NS	**< 0.001**	**< 0.001**	**< 0.001**	**< 0.001**	**< 0.001**	**< 0.001**	**0.001**	**0.051**	**0.002**	**0.008**	**0.060**
Acids source	Ascorbic acid (C)	**0.002**	**0.006**	**0.003**	**0.005**	**0.011**	**0.008**	NS	**< 0.001**	NS	**0.012**	**< 0.001**	**0.034**	**0.028**	**< 0.001**	**0.040**
	Citric acid (B)	NS	NS	NS	**0.029**	**0.024**	**0.044**	NS	NS	NS	NS	NS	NS	NS	NS	NS
aC		NS	NS	**0.024**	NS	**0.005**	**0.015**	NS	NS	NS	NS	NS	NS	**0.002**	**0.001**	NS

	CD	NS	NS	NS	NS	NS	NS	**0.010**	**0.001**	NS	NS	NS	NS	NS	NS	NS
Interaction	aD	NS	NS	NS	NS	NS	NS	NS	NS	NS	NS	NS	NS	NS	NS	NS
	BC	NS	NS	NS	NS	NS	NS	**0.020**	NS	NS	NS	NS	NS	NS	NS	NS
Model R^2		0.53	0.60	0.72	0.47	0.81	0.79	0.80	0.82	0.45	0.67	0.75	0.41	0.69	0.85	0.28

The value in bold number indicate significant model terms; NS indicate insignificant model terms (not counting those required to support hierarchy).
